# Facile Synthesis of a Next Generation Safety‐Catch Acid‐Labile Linker, SCAL‐2, Suitable for Solid‐Phase Synthesis, On‐Support Display and for Post‐Synthesis Tagging

**DOI:** 10.1002/slct.201701519

**Published:** 2017-08-16

**Authors:** Christophe Portal, Martin Hintersteiner, Olivier Barbeau, Peter Dodd, Margaret Huggett, Irene Pérez‐Pi, David Evans, Manfred Auer

**Affiliations:** ^1^ School of Biological Sciences and Edinburgh Medical School: Biomedical Sciences University of Edinburgh, The King's Buildings, Edinburgh Scotland EH9 3BF U.K.; ^2^ Edinburgh BioQuarter 9 Little France Road, Edinburgh Scotland EH16 4UX U.K.

**Keywords:** One-bead one-compound, on-bead screening, Safety catch linker, SCAL linker, solid phase synthesis

## Abstract

The SCAL linker, a safety catch linker, is amongst the most versatile linkers for solid phase synthesis. It was originally described in 1991 by Pátek and Lebl. Yet, its application has been hindered by the low yields of published synthetic routes. Over time, the exceptional versatility of this linker has been demonstrated in several applications of advanced solid phase synthesis of peptides and peptidomimetics. Recently, an updated synthesis of the original linker has also been presented at the 22^nd^ American Peptide Symposium, comprising 10 steps. Herein, the design and synthesis of a next generation SCAL linker, SCAL‐2, is reported. SCAL‐2 features a simplified molecular architecture, which allows for a more efficient synthesis in 8 steps with superior yields. Both linkers, SCAL and SCAL‐2 are compared in terms of their cleavage properties adding valuable information on how to best utilize the versatility of these linkers for solid phase synthesis.

High throughput organic synthesis techniques and especially the use of solid phase chemistry has enabled the cost effective generation of libraries of hundreds to hundreds of thousands of compounds with high purity and drastically shortened production times. Solid phase linkers are cleavable moieties which anchor the product to the solid support during the synthesis and then allow for the selective release of the final compounds. Linkers must therefore remain stable during the synthesis and at the same time be cleavable under mild conditions in order not to degrade the desired product. Since the first use of solid supported synthesis by Merrifield,^1^ many examples of solid phase linkers have been reported, the vast majority of which are cleavable by either electrophilic, nucleophilic, reductive or oxidative conditions.^2^ However, the inherent sensitivity of these linkers to a certain set of cleavage conditions prevents the same conditions from being used in the synthesis of the supported compounds. This has led to entire synthesis schemes being developed for specific linker types with their respective sensitivities. The Fmoc based and Boc based peptide synthesis schemes are prime examples of syntheses schemes being tuned to a specific linker.

Furthermore, in classical solid phase synthesis the final compound is often generated by the simultaneous detachment from solid support and deprotection of various side‐chain protecting groups. However, modern Chemical Biology research, which uses compounds as tools for understating biological systems increasingly utilizes post‐synthesis tagging methods.^3^, ^4^, ^5^, ^6^, ^7^ Also, the use of combinatorial libraries for on‐bead screening^8^, ^9^, ^10^, ^11^, ^12^ or similar display technologies^13^, ^14^, ^15^ requires that the final compounds can be fully deprotected while it still remains linked to the solid surface. These requirements for flexibility and increased chemical compatibility have led to the development of more sophisticated linker concepts, such as photo‐cleavable linkers^16^, ^17^, ^18^ and safety–catch linkers.^19^, ^20^, ^21^ Although providing a high‐degree of chemical orthogonality, photolinkers often suffer from inefficient compound cleavage due to photo‐shielding, the generation of highly reactive intermediates, and their incompatibility with photosensitive tags such as fluorescent dyes. Therefore, the use of safety catch linkers is often preferred.

**Figure 1:**
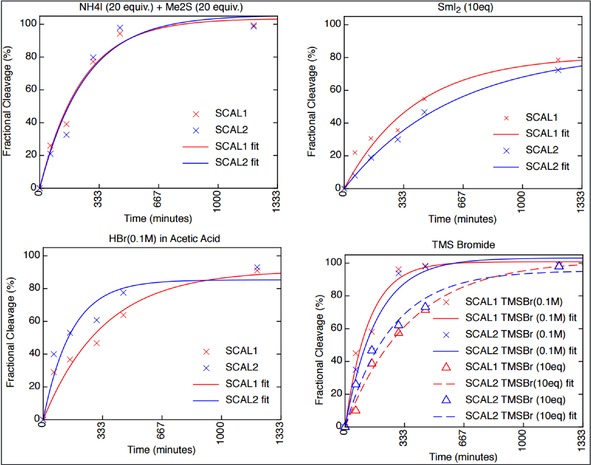
Cleavage kinetics of SCAL‐2 linker (blue) compared to the original SCAL linker (red) under different cleavage conditions. Data were fitted by non‐linear regression assuming a mono‐exponential characteristic.

**Figure 2:**
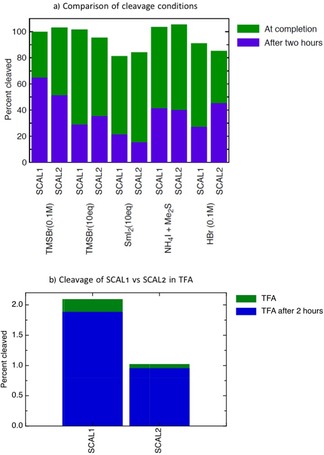
a) top: Cleavage efficiency of the original SCAL 1 linker and the new SCAL‐2 linker under different cleavage conditions. b) bottom: Premature cleavage of SCAL 1 and the new SCAL‐2 linker from resin in their oxidized form (without prior reduction), when treated with TFA. Blue bars denote the fraction of compound cleaved after two hours, green bars, the fraction cleaved at saturation (data from non‐linear curve fits of Figure 1.

Conceptually, the increased stability and cleavage specificity of safety catch linkers is achieved by using a two‐step cleavage procedure: In its non‐activated form, the linker is inert to most common reaction conditions. A simple and specific transformation then activates the linker and makes it susceptible to one of the classical cleavage conditions, such as strong acids, strong bases or nucleophilic cleavage. Common examples of safety catch linkers are the sulfonamide based Kenner and Ellman linkers and derivatives thereof.^22^, ^23^, ^24^ Cleavage of these linkers is carried out by alkylation of the sulphonamide, for example by iodoacetonitrile, which makes the linker sensitive to nucleophiles like NaOH or primary amines. Although being great tools for solid phase synthesis, these linkers do not allow full deprotection of compounds on solid support and post‐synthesis tagging due to the strong alkylating conditions required to trigger the safety‐catch mechanism. Lebl et al. reported the use of a benzhydrylamine acid labile safety catch linker **1** for peptide synthesis.^25^ The linker, depicted in Scheme 1, is based on the original Rink amide linker, making use of methyl thioether substituents instead of the methoxy derivatives. This modification allowed switching off the acid sensitivity of the linker by oxidising the sulphur atoms. For the first time this provided a linker which offers full stability under the most common synthesis and cleavage conditions including acid, base, nucleophilic conditions, etc, yet, being cleavable under relatively mild conditions. A set of reductive acidic treatments was shown to cleave the desired material from the solid phase. This first safety catch linker has been used for its stability and versatility by many research groups for a variety of applications, e. g. chemical ligations^26^,^27^, glycoconjugate^28^ and glycopeptide^29^ syntheses and cyclizations.^30^ Similar thioether/sulfone based linkers as well as seminal examples of other safety catch linkers have been reported to date.^31^, ^32^, ^33^ Most reported examples are quite specific to a family of synthesis products and do not appear as versatile as Lebl's SCAL linker **1** (Scheme 1).

**Scheme 1:**
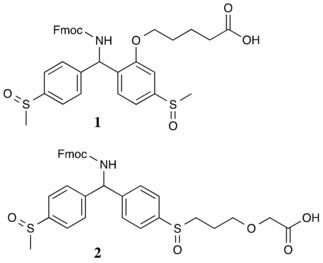
Lebl's SCAL linker (top) and the new SCAL‐2 linker (bottom).

Although a chemically elegant solution, the main drawback of the SCAL linker is its cumbersome 14‐step synthesis and the low overall yields of the original synthetic route. This limitation was also acknowledged by the original inventors and recently addressed in a presentation at the 22nd American Peptide Symposium, however without a follow‐up publication including full experimental details to‐date.^34^ The key step in the originally published synthesis of the SCAL linker is the Friedel‐Crafts acylation, which builds up the benzophenone core of the linker but results in notoriously low yields, due to ambiguous regio‐directing effects of the methyl thioether and the methyl ether groups, the latter being required as attachment functionality for anchoring the linker onto the solid support.

To overcome this strategic weakness, we designed SCAL‐2, **2**, where the separate ether attachment point is eliminated and the anchoring carboxylic acid grafted onto one of the two thioether substituents, thereby simplifying the molecule for more efficient synthesis.

The synthesis of the benzhydrylamine linker **2** was realised, as described in Scheme 2, by the reduction of a key ketoxime intermediate **3**, prepared from the corresponding Friedel‐Crafts benzophenone **4**. As the tert‐butyl‐diphenyl‐silane protecting group showed partial cleavage during the key Friedel‐Crafts step the crude mixture was treated with TBAF after standard workup without isolating the intermediate. The dialkylsulfanyl‐benzophenone **4** obtained after acylation of **5** by 4‐methylthiobenzoyl chloride **6** was subsequently alkylated by tert‐butyl‐bromoacetate to afford **7**. This route was chosen to afford the required carboxylic acid functionality, after miscellaneous attempts to oxidise the primary alcohol had failed. Finally, the free amino compound **8** obtained by the reduction of the oxime functionality was oxidised into **9** and readily Fmoc protected to give compound **10** and the tert‐butyl group removed to yield the desired amino acid linker **2**. Thus, the target molecule SCAL‐2 was synthesized in a total of 8 steps with excellent yields. The Friedel‐Crafts acylation needed substantial optimisation. At first, the synthesis was attempted with tert‐butyl‐dimethyl‐silyl protection but the protecting group showed partial cleavage during the Friedel‐Crafts step in preliminary experiments. In an attempt to solve this problem, the more stable tert‐butyl‐diphenyl‐silyl group was employed, but it also showed partial cleavage during the Friedel‐Crafts reaction. Therefore the strategy chosen was to first fully deprotect the crude mixture of the Friedel‐Crafts reaction, which after work‐up gave **4** in 70 % yield over 2 steps, followed by introduction of a Boc protecting group on the side chain anchoring point.

**Scheme 2:**
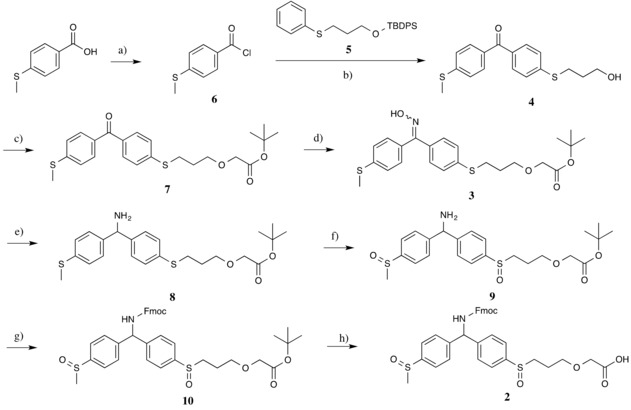
Synthesis of new SCAL linker. *Reagents and conditions*; (a) SOCl_2_ (3 equiv.), DCE, 65 °C, 3 h, quant.; (b) (1), **5**, AlCl_3_ (1.4 equiv.), DCM, 0 °C, 1hr then **6** added, 0 °C for 5 h, 6 N HCl; (2) TBAF (1.2 equiv.), THF, 0 °C then RT, 1 h, 70 % over 2 steps; (c) *t*‐butyl bromoacetate (3 equiv.), NBu_4_Br (3.5 mol%), 50 % NaOH, toluene, RT, 73 %; (d) NH_2_OH.HCl (5 equiv.), NaOAc (10 equiv.), EtOH, reflux, 15 h, 66 %; (e) Zn (10 equiv.), AcOH, RT, 30 min, 80 %; (f) NaIO_4_, MeOH, H_2_O, 5 °C to RT, 15 h, 89 %; (g) FmocOSu (1.2 equiv.), NaHCO_3_ (1.2 equiv.), H_2_O in MeCN (1:4), 24 h, 62 %; (h) TFA in DCM (1:5 v/v), 24 h, 99 %.

The strategy used for the synthesis of linker **2** overcomes several of the disadvantages of the original approach. First, the synthesis is six steps shorter. In addition, our version of the reaction procedure moves the yield limiting Friedel‐Crafts acylation step earlier in the synthesis, therefore limiting the loss of material at later stages. Furthermore, the removal of the additional ether substitution on the benzhydrylamine core reduces the electron density in the conjugated system and should further stabilize the linker in its non‐activated form.

After stepwise optimization of the production of SCAL‐2, the cleavage properties of the two linkers were compared. Therefore, the Fmoc protected linker **2** was immobilised onto TentaGel resin, followed by Fmoc‐Phe‐OH after Fmoc group deprotection. A similar Fmoc‐Phe‐OH resin was also prepared using the original SCAL linker **1**. Both resins were then subjected to different cleavage reaction mixes and the release of Fmoc‐Phe‐NH_2_ was evaluated by HPLC analysis and comparison of the peak area value with a previously obtained standard concentration curve. A range of commonly used mixtures for the cleavage of the SCAL linker were used, such as TFA; TMSBr (0.1 M) in TFA; TMSBr (10 equiv. relative to resin) in TFA; SmI_2_ (10 equiv. relative to resin) in TFA; NH_4_I (20 equiv. relative to resin), Me_2_S (20 equiv. relative to resin) in TFA; HBr in acetic acid (1:3 w/v).^35^, ^36^, ^37^, ^38^ Aliquots of the deprotection reactions were collected at regular times (60 min, 150 min, 300 min, 450 min, 1200 min) and replaced with the same volume of fresh cleavage reaction mixtures.

Both linkers, the original SCAL linker and the new SCAL‐2 linker, exhibited very similar stabilities during these cleavage studies (Figure 1). The kinetics profiles however, revealed significant differences with respect to the efficiency of the different cleavage reaction cocktails. The best results were obtained with TMSBr in TFA (0.1 M), with essentially 100 % cleavage efficiency after 450 min. Samarium Iodide on the other hand resulted in only moderate overall cleavage yields for both linkers, SCAL‐1 and SCAL‐2.

Furthermore, the kinetics data were analysed for the fraction of compound cleaved after two hours and at completion for each linker (Figure 2 **a**). In this head‐to‐head comparison, the new linker, SCAL‐2, tends to require slightly longer exposure times compared to the original SCAL linker, while showing the same yields of cleaved compound at saturation. This trend was predictable from the structural differences that exist between the two linkers. The more electron rich SCAL linker core increases the stability of the reactive intermediate, thereby increasing slightly the speed of the cleavage reaction. More notably, however, the direct corollary to this is that the original SCAL linker is more prone to cleavage also in its oxidised (sulphoxide) form. Experiments for testing pre‐mature cleavage using typical BOC‐cleavage conditions, resulted in twice as much leakage of compound from resin containing the original SCAL linker as compared to SCAL‐2 (Figure 2 **b**). Such differences in linker stability can lead to significant yield improvements and purity of final compounds during longer solid phase syntheses, involving repetitive TFA cleavage steps. A further stabilization might be achievable with additional deactivating groups on the benzophenone core. In summary, we have developed a new safety catch acid labile linker, SCAL‐2, with simplified molecular architecture, easier chemical accessibility and improved stability. The preparation of the compound is achieved in 8 linear steps and in good overall yields, suitable for scale‐up and large scale synthesis. SCAL‐2 **2** is fully characterized (see supplementary information), and allows Fmoc and/or Boc strategies to be employed for peptide or small molecule synthesis.

## Supporting Information Summary

Supporting information containing detailed synthetic conditions and characterization of intermediates **3‐10** and final compound **2** (LC–MS, NMR) is available.

## Conflict of interest

The authors declare no conflict of interest.

## Supporting information

As a service to our authors and readers, this journal provides supporting information supplied by the authors. Such materials are peer reviewed and may be re‐organized for online delivery, but are not copy‐edited or typeset. Technical support issues arising from supporting information (other than missing files) should be addressed to the authors.

Supplementary
