# Supplementary material for: Facile Synthesis of a Next Generation Safety‐Catch Acid‐Labile Linker, SCAL‐2, Suitable for Solid‐Phase Synthesis, On‐Support Display and for Post‐Synthesis Tagging
Source: ChemistrySelect. 2017 Aug 16;2(23):6658–62. doi: 10.1002/slct.201701519 (PMC5661701; doi:10.1002/slct.201701519)
Supplement: Supplementary file 1 — Supplementary [file SLCT-2-6658-s001.pdf]

Chemistry SELECT

Supporting Information

© Copyright Wiley-VCH Verlag GmbH & Co. KGaA, 69451 Weinheim, 2017

Facile Synthesis of a Next Generation Safety-Catch Acid-Labile Linker, SCAL-2, Suitable for Solid-Phase Synthesis, On-Support Display and for Post-Synthesis Tagging

Christophe Portal, Martin Hintersteiner, Olivier Barbeau, Peter Dodd, Margaret Huggett, Irene Pérez-Pi, David Evans, and Manfred Auer*

Electronic Supplementary Information

Contents

- General
- Procedures
- References
- ^{1}H and ^{13}C NMR spectra

General

^{1}H and ^{13}C NMR were recorded on a Bruker Avance III spectrometer at 400/500/90/126 MHz respectively. Chemical shifts (δ) are reported in ppm in the solvents specified. High-resolution mass spectrometry (HRMS) was performed using a 12T SolariX FT-ICR (Bruker Daltonics) equipped with ESI. APCI LC/MS spectra were recorded on a Thermo Finnigan LCQ Deca XP. Silica gel Si-60 (40-63 µm), or Biotage® KP-Sil Zip® Columns were used for flash chromatography using the specified solvents. Solvents were obtained commercially in anhydrous form, and not distilled before use. Thin-layer chromatography was performed on aluminium-backed pre-coated Merck silica gel (60 F254) plates, and compounds were visualized using UV light and/or iodine vapour.

Procedures

4-methylsulfanylbenzoyl chloride (6)

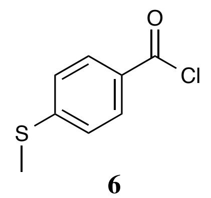

6

Material prepared by published method [1] or purchased from Sigma Aldrich.

[4-(3-hydroxypropylsulfanyl)phenyl]-(4-methylsulfanylphenyl)methanone (4)

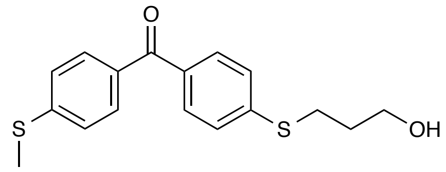

4

4-methylsulfanylbenzoyl chloride (15.5g, 0.083mol) was dissolved in dry DCM (250ml), under N_2, and cooled to 0^oC. $AlCl_3$ (15.5g, 0.116 mol, 1.4eq), which had been ground and dried under vacuum at 50^oC, was added and the resulting orange coloured solution stirred for 1h at 0^oC. The tert-butyl-diphenyl-(3-phenylsulfanylpropoxy)silane (34g, 0.083mol) in DCM (50ml) was added over 20 minutes at 0^oC and then the RM was stirred at 0^oC for 5h. TLC (SiO_2, EtOAc:Cyclohexane 1:1, UV visualisation) shows SM consumed. The RM was quenched with 80ml of 6N HCl and stirred overnight at RT. The RM was diluted with water and DCM and separated. After two further extractions with DCM the combined organics were washed with water, dried ($MgSO_4$) and evaporated to dryness. The yellow oil obtained was taken up in THF (200ml), cooled to 0^oC and the TBAF (1M in THF, 100ml, 0.1mol) was added. The RM was allowed to warm up to RT and after 2h TLC showed complete reaction. The RM was concentrated and then partitioned between water and Et_2O. After two further extractions with Et_2O the combined organics were washed with brine, dried ($MgSO_4$) and evaporated to dryness. The crude product was purified by flash vacuum chromatography on a large pad of SiO_2, eluting with 50-100% EtOAc in cyclohexane. This yielded the product as a pale yellow solid, 19g, 70% over two steps.

LC/MS: *m/z* calculated for $C_{17}H_{18}O_2S_2$ $[M + H]^+$ 319.08, found 319.18

^1H NMR (500 MHz, CHLOROFORM-d) δ 7.64-7.75 (m, 4H), 7.33-7.38 (m, 2H), 7.26-7.31 (m, 2H), 3.76-3.84 (m, 2H), 3.14 (t, *J*=7.25 Hz, 2H), 2.52-2.58 (m, 3H), 2.51-2.52 (m, 1H), 1.92-2.00 (m, 2H)

^{13}C NMR (126 MHz, CHLOROFORM-d) δ 195.0, 145.2, 143.7, 134.2, 133.7, 130.4, 126.3, 124.9, 60.9, 31.5, 28.5, 26.9, 14.7

tert-butyl-dimethyl-(3-phenylsulfanylpropoxy)silane (5)

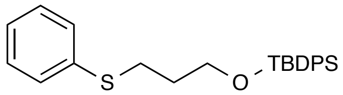

5

Material prepared by published methods [2] via 3-bromopropoxy-tert-butyl-diphenyl-silane, and [3].

[4-[3-[tert-butyl(diphenyl)silyl]oxypropylsulfanyl]phenyl]-(4-methylsulfanylphenyl)methanone (7)

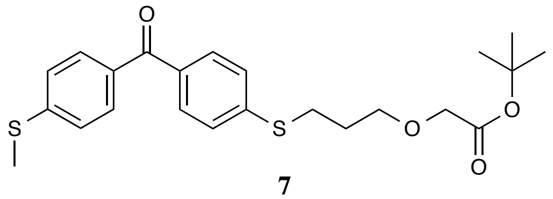

7

[4-(3-hydroxypropylsulfanyl)phenyl]-(4-methylsulfanylphenyl)methanone) (17g, 0.053mol) was dissolved in toluene (80ml), Bu_4NBr (600mg, 1.8mmol) and tert-butyl bromoacetate (15.6ml, 0.106mol, 2eq) were added, followed by 50% NaOH (50ml). Reaction was then stirred vigorously for 4h. TLC (SiO_2, EtOAc:cyclohexane 1:4) showed complete reaction. Reaction was partitioned between water and toluene. The aqueous layer was extracted twice more with toluene and then the combined organic layers were washed with water and brine, dried ($MgSO_4$) and evaporated to dryness, yielding a pale yellow gum, 17g, 73%.
200 mg crude material chromatographed, Biotage® Zip® Cartridge 5g. Eluted with Cyclohexane/EtOAc 9/1
LC/MS: *m/z* calculated for $C_{23}H_{28}O_4S_2$ $[M + H]^+$ 433.15, found 433.01; calculated for $[M + Na]^+$ 455.13, found 455.15
^1H NMR (500 MHz, CHLOROFORM-d) δ 7.65-7.79 (m, 4H, C(O)CC$\mathbf{H}_{\mathbf{aro}}$), 7.36-7.41 (m, 2H, $CH_3SCC\mathbf{H}_{\mathbf{aro}}$), 7.30-7.34 (m, 2H, $CH_2SCC\mathbf{H}_{\mathbf{aro}}$), 3.98 (s, 2H, $OC\mathbf{H_2}C(O)$), 3.68 (t, *J*=5.99 Hz, 2H, $SC\mathbf{H_2}CH_2CH_2$), 3.18 (t, *J*=7.25 Hz, 2H, $SCH_2CH_2C\mathbf{H_2}$), 2.57 (s, 3H, $C\mathbf{H_3}S$), 1.98-2.08 (m, 2H, $CH_2C\mathbf{H_2}CH_2$), 1.46-1.53 (m, 9H, $(C\mathbf{H_3})_3$).
^{13}C NMR (126 MHz, CHLOROFORM-d) δ 194.9, 169.6, 145.0, 143.4, 134.4, 133.9, 130.5, 126.5, 124.9, 81.7, 69.7, 68.9, 29.2, 28.8, 28.1, 14.9

tert-butyl 2-[3-[4-[N-hydroxy-C-(4-methylsulfanylphenyl)carbonimidoyl]phenyl]sulfanylpropoxy]acetate (3)

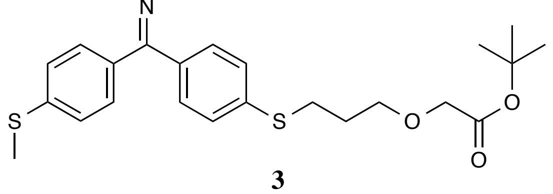

3

[4-[3-[tert-butyl(diphenyl)silyl]oxypropylsulfanyl]phenyl]-(4-methylsulfanylphenyl)methanone (**7**, 38g, 0.088mol) was mixed in EtOH (500ml) and the hydroxylamine hydrochloride (31g, 0.44mol) and sodium acetate (72g, 0.88mol) were added.

The RM was heated at reflux overnight. TLC (EtOAc:cyclohexane 1:3) showed complete reaction. The RM was cooled, filtered and concentrated. The residue was partitioned between water and EtOAc. After 2 further extractions the combined organics were washed with brine, dried ($MgSO_4$) and evaporated. The crude product was purified on a large SiO_2 pad, eluting with EtOAc:cyclohexane 1:3. The product was isolated as a yellow gum, 26g (66%). Material was used without further purification.
LC/MS: *m/z* calculated For $C_{23}H_{29}NO_4S_2$ $[M + H]^+$ 448.16, found 448.00

tert-butyl 2-[3-[4-[amino-(4-methylsulfanylphenyl)methyl]phenyl]sulfanylpropoxy]acetate (8)

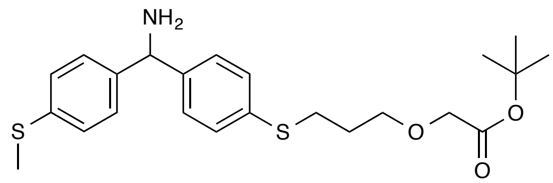

8

tert-butyl 2-[3-[4-[N-hydroxy-C-(4-methylsulfanylphenyl)carbonimidoyl]phenyl]sulfanylpropoxy]acetate (1 g, 2.24 mmol) was dissolved in AcOH . Zn (1.46 g, 22.36 mmol) was added with ice cooling. After 30 minutes the reaction was complete, and the solvent was removed *in vacuo*, and azeotroped with toluene twice, to give an amber gum
LC/MS: *m/z* calculated for $C_{23}H_{31}NO_3S_2$ $[M + H]^+$ 434.18, found 434.11; calculated for $[M + Na]^+$ 456.16, found 456.23
^{1}H NMR (500 MHz, CHLOROFORM-d) δ 7.20-7.35 (m, 8H), 5.09-5.27 (m, 1H), 3.95 (s, 2H), 3.63 (t, *J*=6.15 Hz, 2H), 3.04 (t, *J*=7.09 Hz, 2H), 2.48 (s, 3H), 1.94 (t, *J*=7.09 Hz, 2H), 1.44-1.55 (m, 9H).
^{13}C NMR (126 MHz, CHLOROFORM-d) δ 169.7, 137.1, 135.0, 129.4, 127.4, 126.9, 81.6, 69.8, 68.9, 58.8, 30.3, 29.4, 28.1, 16.0

[4-[amino-[4-[3-(2-tert-butoxy-2-oxo-ethoxy)propyl-oxido-sulfonio]phenyl]methyl]phenyl]-methyl-oxido-sulfonium (9)

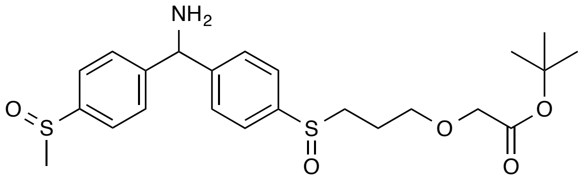

9

tert-butyl 2-[3-[4-[amino-(4-methylsulfanylphenyl)methyl]phenyl]sulfanylpropoxy]acetate (22g, 0.05mol) was mixed in MeOH (150ml) and cooled in an ice-bath. Sodium periodate (21.4g, 0.1mol) was dissolved in water (50ml) and then added over 1h to the RM. The RM was allowed to warm up to RT and then stirred at RT overnight. TLC (SiO_2, 5% MeOH in DCM) showed reaction complete. Reaction mixture was filtered through Celite to remove all inorganic salts. The filtrate was concentrated and then partitioned between water and DCM. The combined organic washes were washed with brine, dried over $MgSO_4$ and evaporated to dryness, yielding the product as an orange coloured gum, 21g, 89%.

LC/MS: *m/z* calculated for $C_{23}H_{31}NO_5S_2$ $[M + H]^+$ 466.17, found 465.97

^{1}H NMR (500 MHz, CHLOROFORM-d) δ 7.46-7.68 (m, 8H), 5.34 (s, 1H), 3.91 (s, 2H), 3.53-3.72 (m, 2H), 3.02 (ddd, *J*=6.15, 9.46, 13.40 Hz, 1H), 2.86 (ddt, *J*=1.26, 4.73, 8.99 Hz, 1H), 2.71 (s, 3H), 2.12-2.21 (m, 2H), 1.99-2.13 (m, 2H), 1.91 (ddd, *J*=4.89, 7.33, 9.54 Hz, 1H), 1.38-1.54 (m, 9H)

^{13}C NMR (126 MHz, CHLOROFORM-d) δ 169.5, 148.1, 147.9, 144.6, 142.7, 127.9, 127.8, 124.5, 124.0, 81.7, 69.7, 68.6, 59.2, 54.0, 43.9, 28.1, 22.6

3-(2-tert-butoxy-2-oxo-ethoxy)propyl-[4-[(9H-fluoren-9-ylmethoxycarbonylamino)-[4-[methyl(oxido)sulfonio]phenyl]methyl]phenyl]-oxido-sulfonium (10)

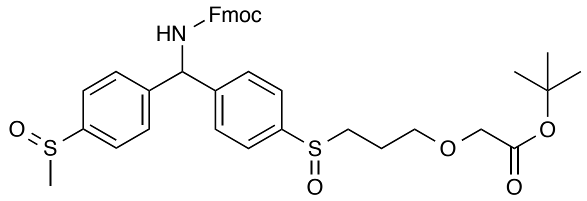

10

[4-[amino-[4-[3-(2-tert-butoxy-2-oxo-ethoxy)propyl-oxido-sulfonio]phenyl]methyl]phenyl]-methyl-oxido-sulfonium (**9**, 20g, 0.043mol) was mixed in water in MeCN (300ml, 1:5 v/v). NaHCO3 (4.3g, 0.0516mol, 1.2eq) and FmocOSu (17.4g, 0.0516mol, 1.2eq) added. Reaction was stirred at RT overnight. Solvent concentrated *in vacuo* and the residue partitioned between water and DCM. The combined organics were washed with water, dried over $MgSO_4$ and evaporated to dryness. The crude product was purified on a large SiO_2 pad, eluting with 0-5% MeOH in DCM. The product was further purified with 0-2% MeOH in EtOAc, yielding the product as a clear foam, 18.5g (62%)

LC/MS: *m/z* calculated for $C_{38}H_{41}NO_7S_2$ $[M + H]^+$ 688.24, found 687.96

^{1}H NMR (500 MHz, CHLOROFORM-d) δ 7.27-7.88 (m, 16H), 6.08 (br. s., 1H), 5.50 (br. s., 1H), 4.57 (d, *J*=6.31 Hz, 2H), 4.24 (br. s., 1H), 3.94 (m, 2H), 3.53-3.76 (m, 2H), 2.81-3.17 (m, 2H), 2.75 (s, 3H), 1.87-2.24 (m, 2H), 1.65 (br. s., 2H), 1.43-1.53 (s, 9H)

3-(carboxymethyloxy)propyl-[4-[(9H-fluoren-9-ylmethoxycarbonylamino)-[4-[methyl(oxido)sulfonio]phenyl]methyl]phenyl]-oxido-sulfonium (2)

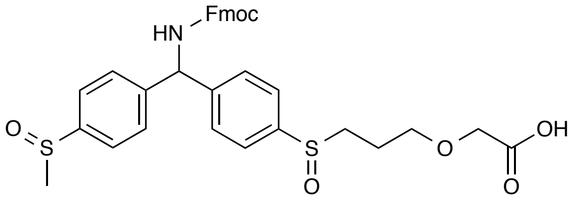

2

3-(2-tert-butoxy-2-oxo-ethoxy)propyl-[4-[(9H-fluoren-9-ylmethoxycarbonylamino)-[4-[methyl(oxido)sulfonio]phenyl]methyl]phenyl]-oxido-sulfonium (**10**, 18.5g, 0.027mol) was dissolved in DCM (100ml) and TFA (20ml) added. The RM was stirred at RT overnight. LCMS showed complete reaction. The reaction mixture was evaporated to dryness and then triturated with Et_2O, yielding a white solid.
HRMS *m/z* (ESI) calculated for $C_{34}H_{33}NO_7S_2$ $[M+H]^+$ 632.1771, found 632.1770.
^{1}H NMR (500 MHz, CHLOROFORM-d) d 7.74-7.83 (m, 2H), 7.56-7.68 (m, 6H), 7.29-7.45 (m, 8H), 6.20 (d, *J*=7.25 Hz, 1H), 6.08 (br. s., 1H), 5.64 (br. s., 1H), 4.53 (br. s., 2H), 4.22 (d, *J*=6.94 Hz, 1H), 3.97-4.10 (m, 2H), 3.62 (br. s., 2H), 3.07 (td, *J*=6.98, 13.48 Hz, 1H), 2.86-3.00 (m, 1H), 2.74 (s, 3H), 1.93-2.17 (m, 2H)
^{13}C NMR (126 MHz, CHLOROFORM-d) d 141.4, 128.4, 127.1, 125.0, 124.8, 124.3, 120.1, 69.6, 68.0, 66.8, 47.3, 43.6

References

[S1] E. Alcalde, N. Mesquida , C. Alvarez-Rúa, R. Cuberes, J. Frigola and S. García-Granda, *Molecules* **2008**, *13*(2), 301-318
[S2] D. Zurwerra, F. Glaus, L. Betschart, J. Schuster, J. Gertsch
W. Ganci, and K.H. Altmann, *Chem. Eur. J.* 2012, 18, 16868 – 16883
[S3] M. Ihara, S. Suzuki , T. Taniguchi, Y. Tokunaga, and K. Fukumoto Tetrahedron Vol. 51, No. 36, pp. 9873-9890. 1995

NMR Spectra

[4-(3-hydroxypropylsulfanyl)phenyl]-(4-methylsulfanylphenyl)methanone (4)

^{1}H NMR

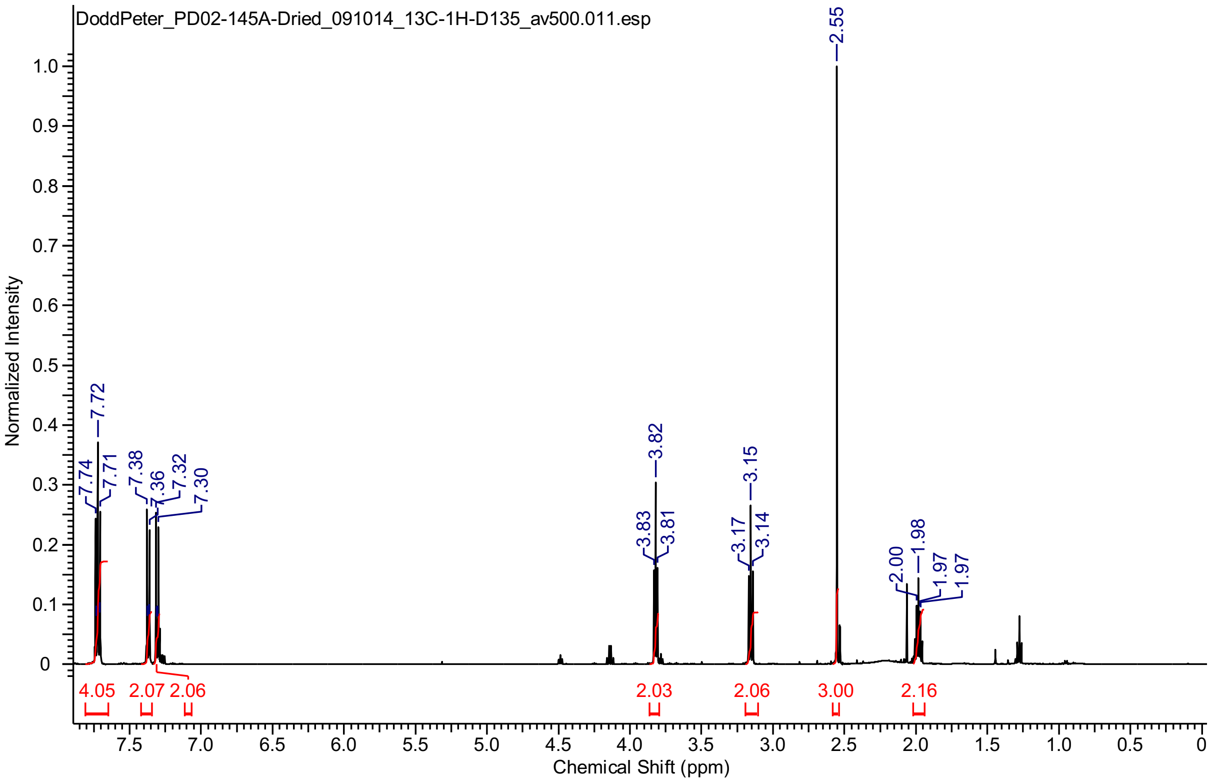

^{13}C NMR

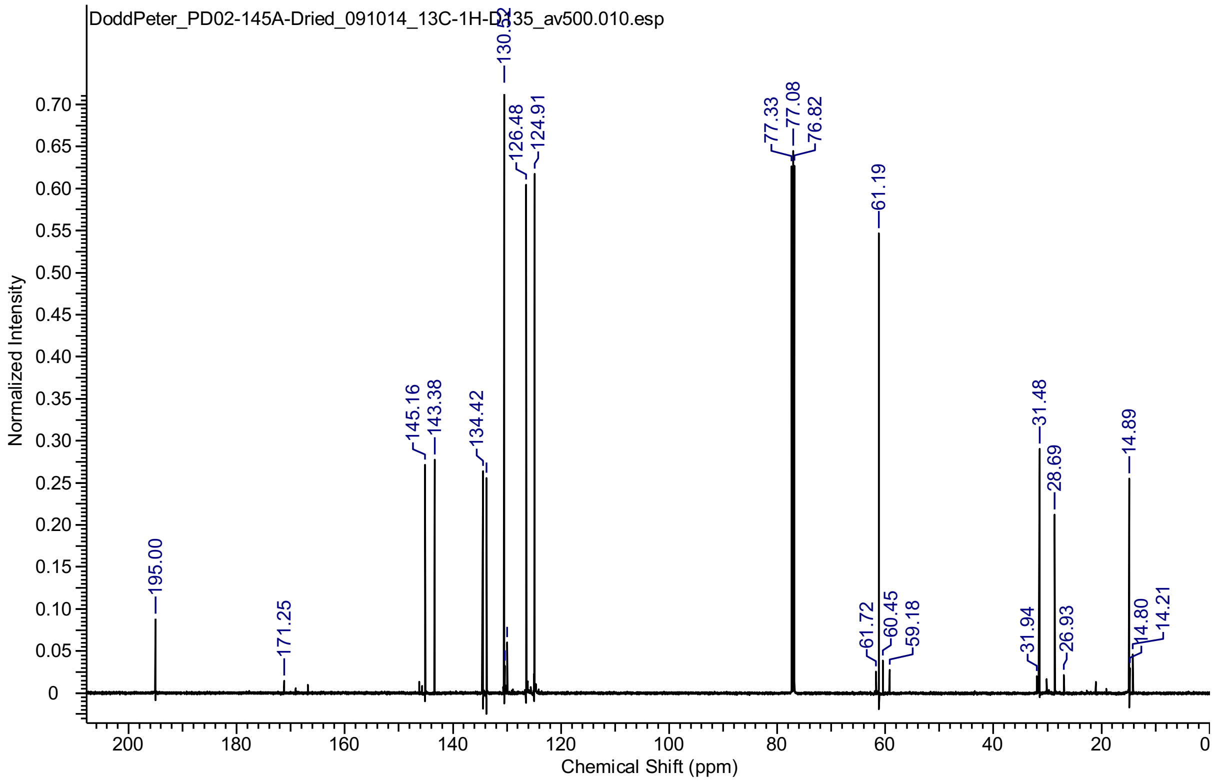

[4-[3-[tert-butyl(diphenyl)silyl]oxypropylsulfanyl]phenyl]-(4-methylsulfanylphenyl)methanone (7)

^{1}H NMR

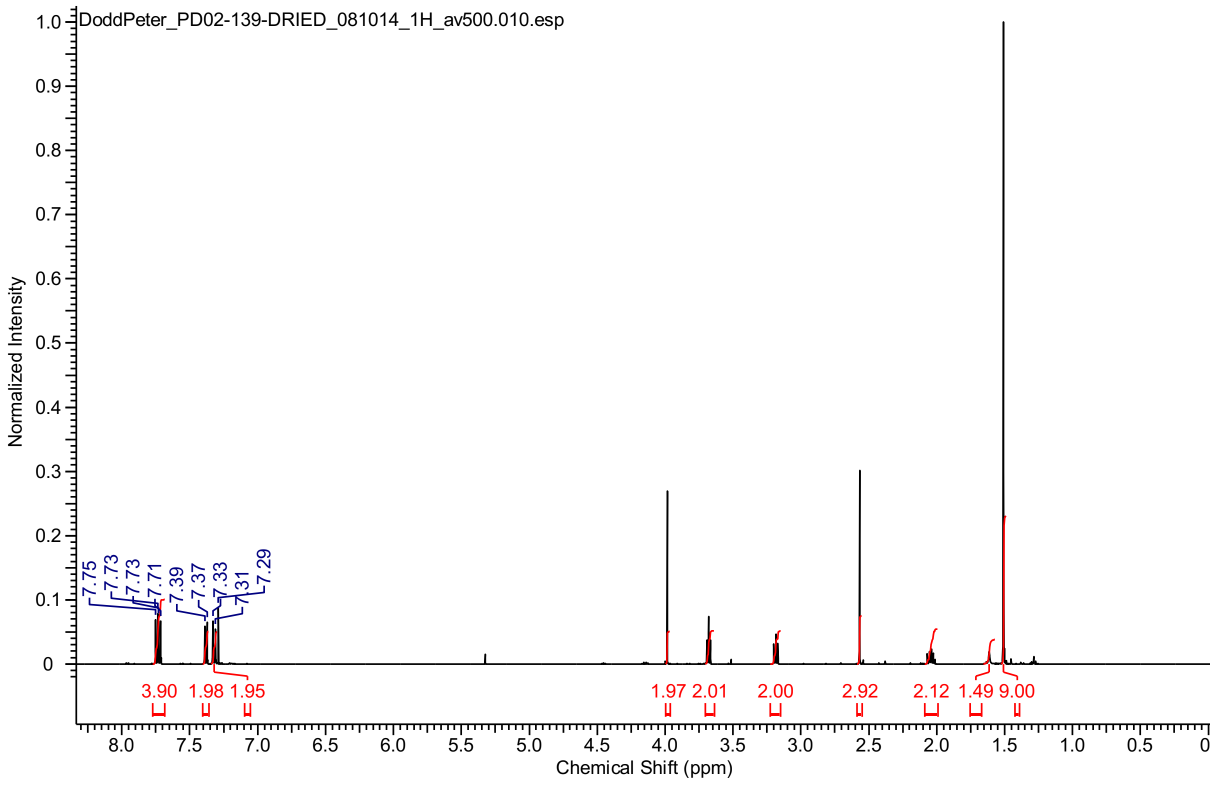

^{13}C NMR

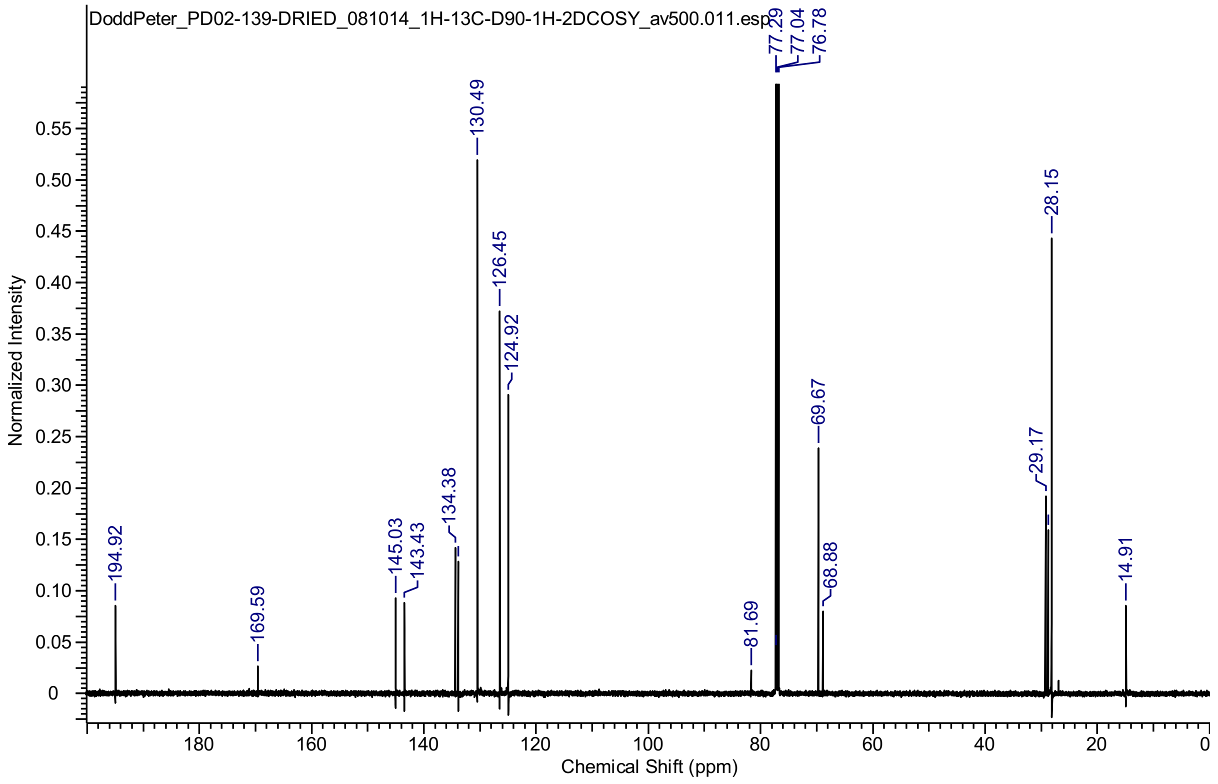

Tert-butyl 2-[3-[4-[amino-(4-methylsulfanylphenyl)methyl]phenyl]sulfanylpropoxy]acetate (8)

^{1}H NMR

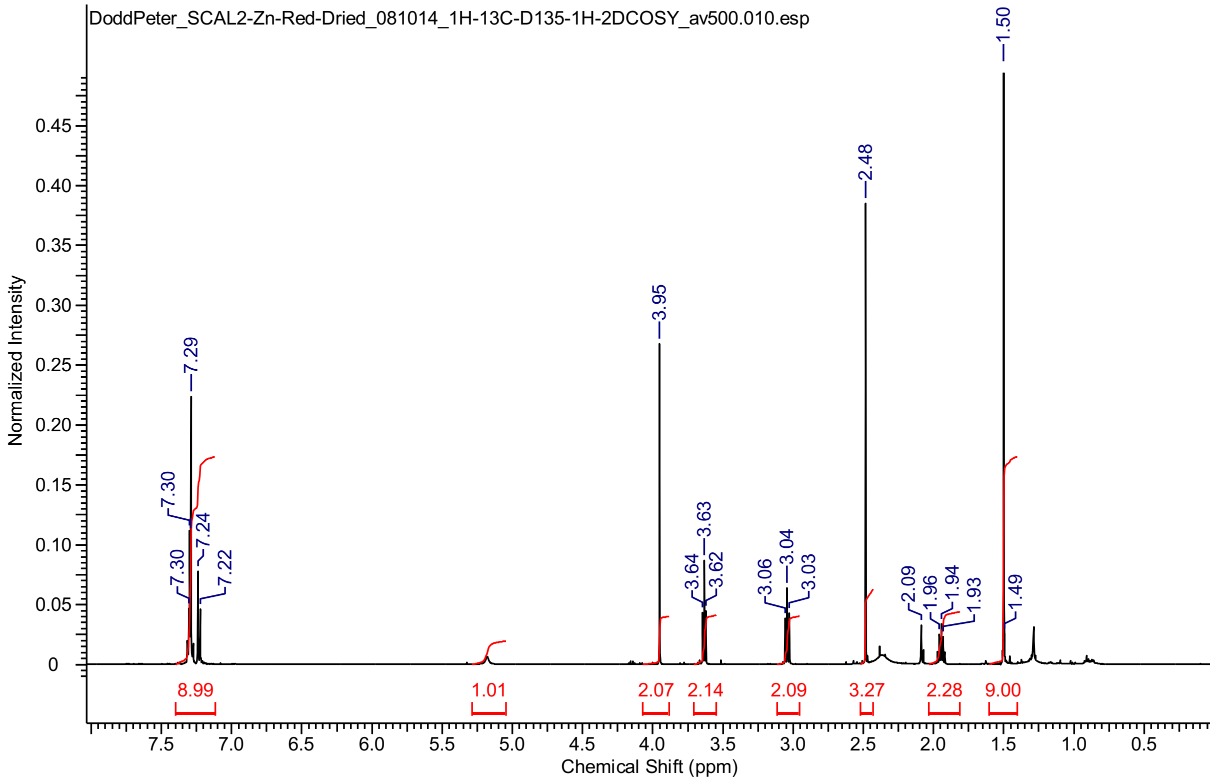

^{13}C NMR

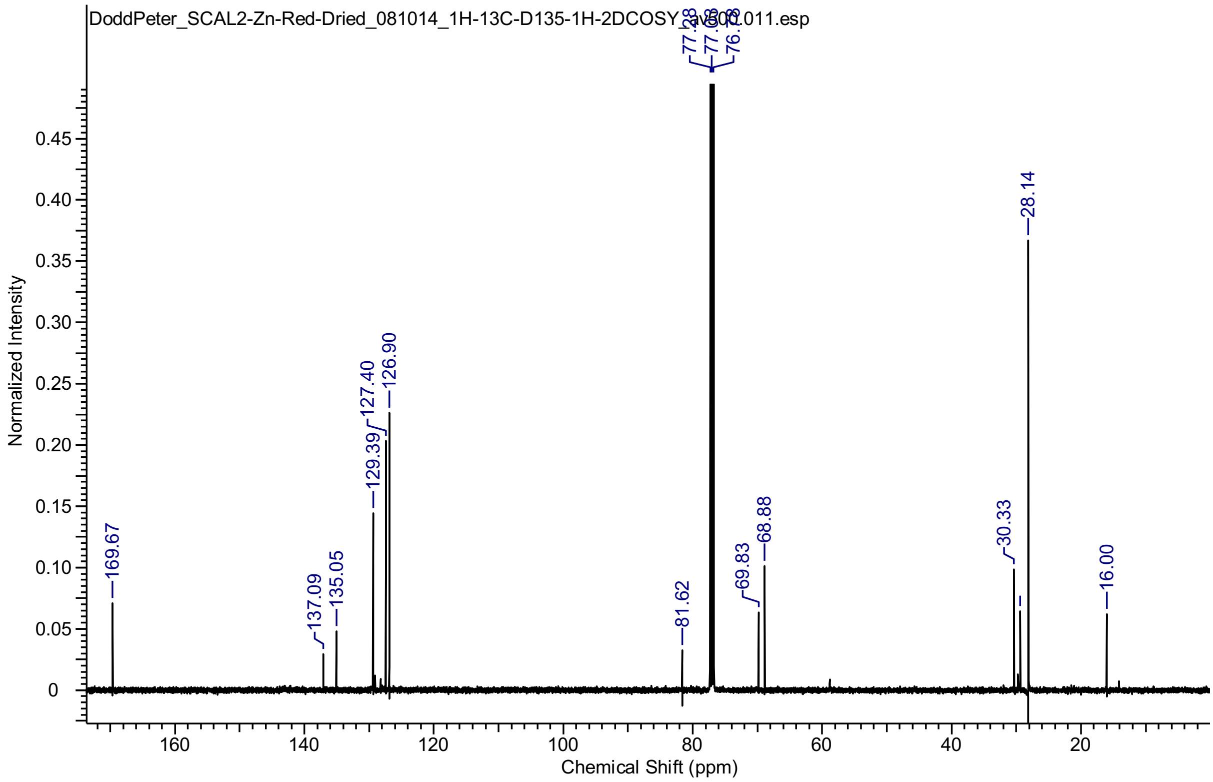

[4-[amino-[4-[3-(2-tert-butoxy-2-oxo-ethoxy)propyl-oxido-sulfonio]phenyl]methyl]phenyl]-methyl-oxido-sulfonium (9)

^{1}H NMR

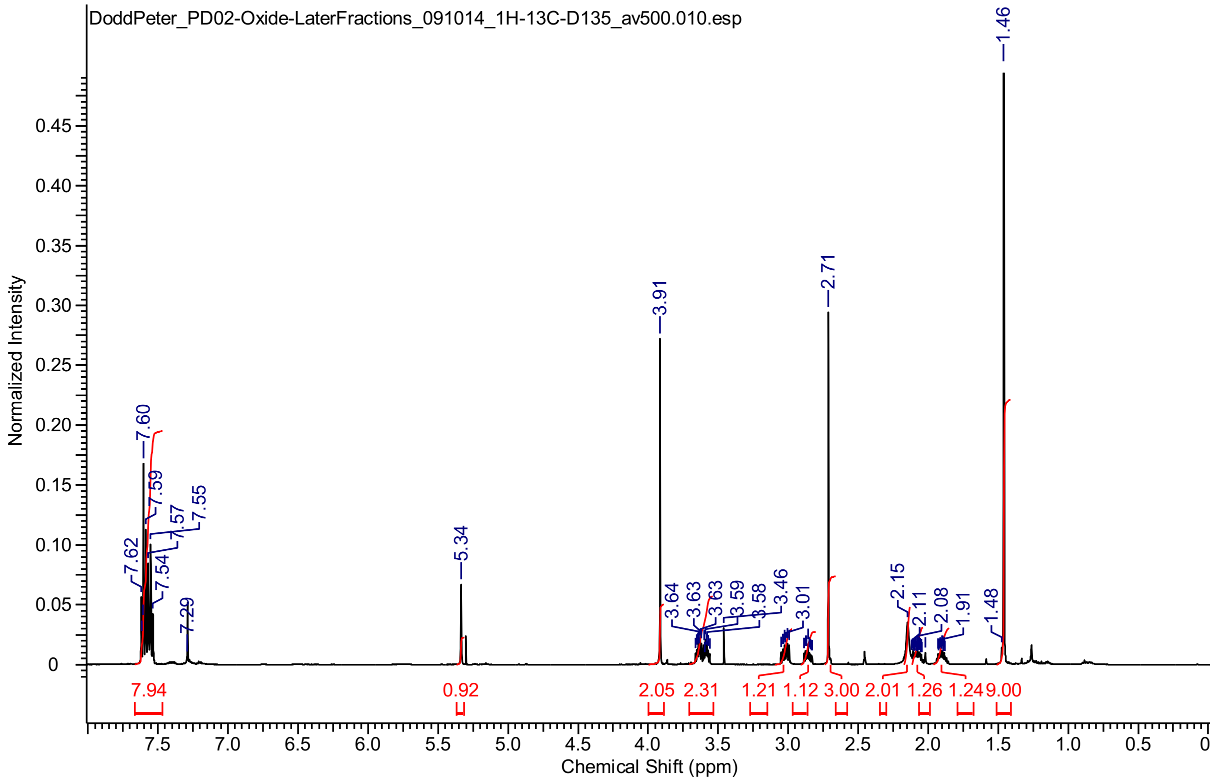

^{13}C NMR

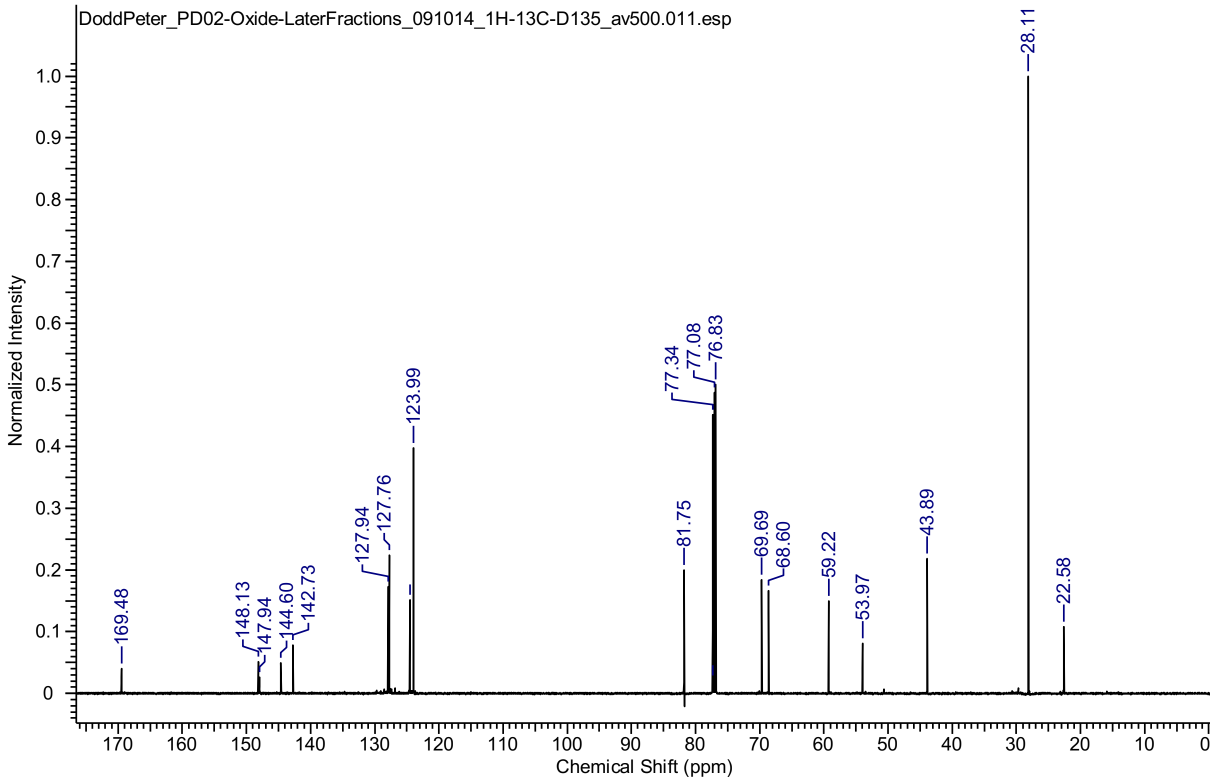

3-(2-tert-butoxy-2-oxo-ethoxy)propyl-[4-[(9H-fluoren-9-ylmethoxycarbonylamino)-[4-[methyl(oxido)sulfonio]phenyl]methyl]phenyl]-oxido-sulfonium (10)

^{1}H NMR

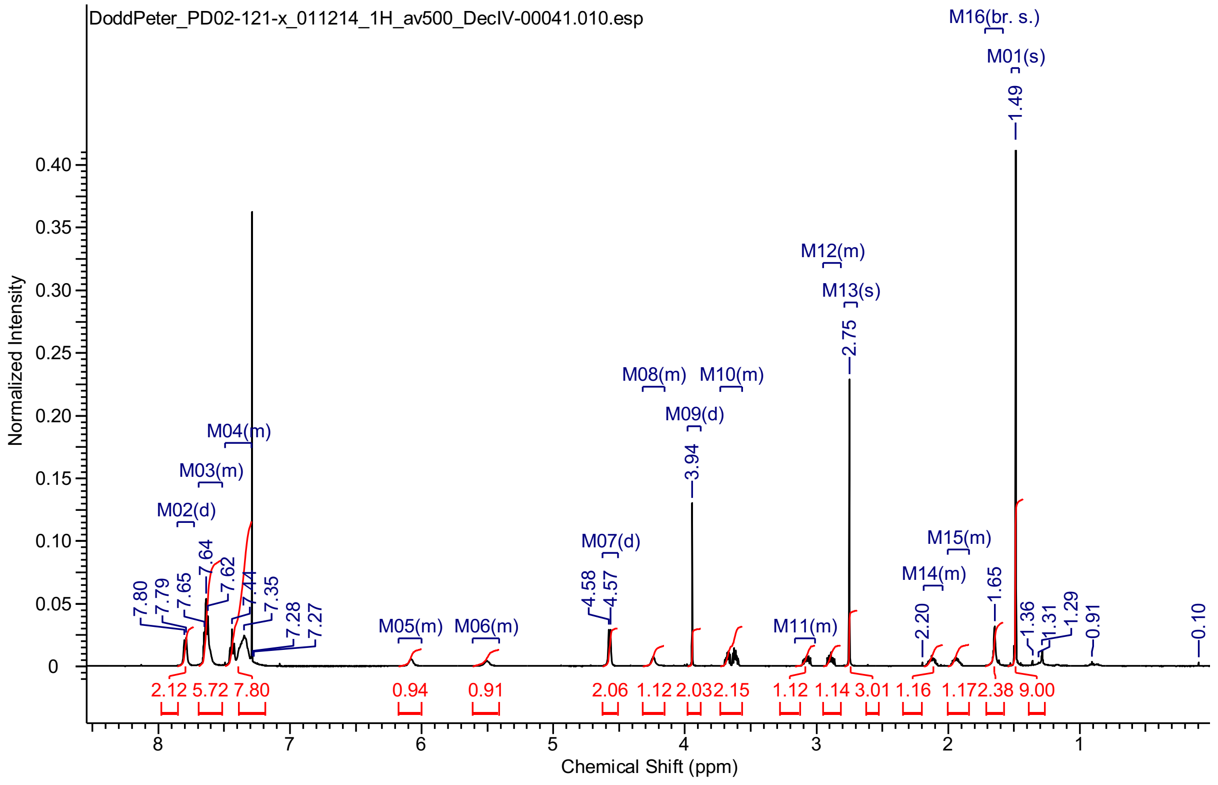

^{13}C NMR

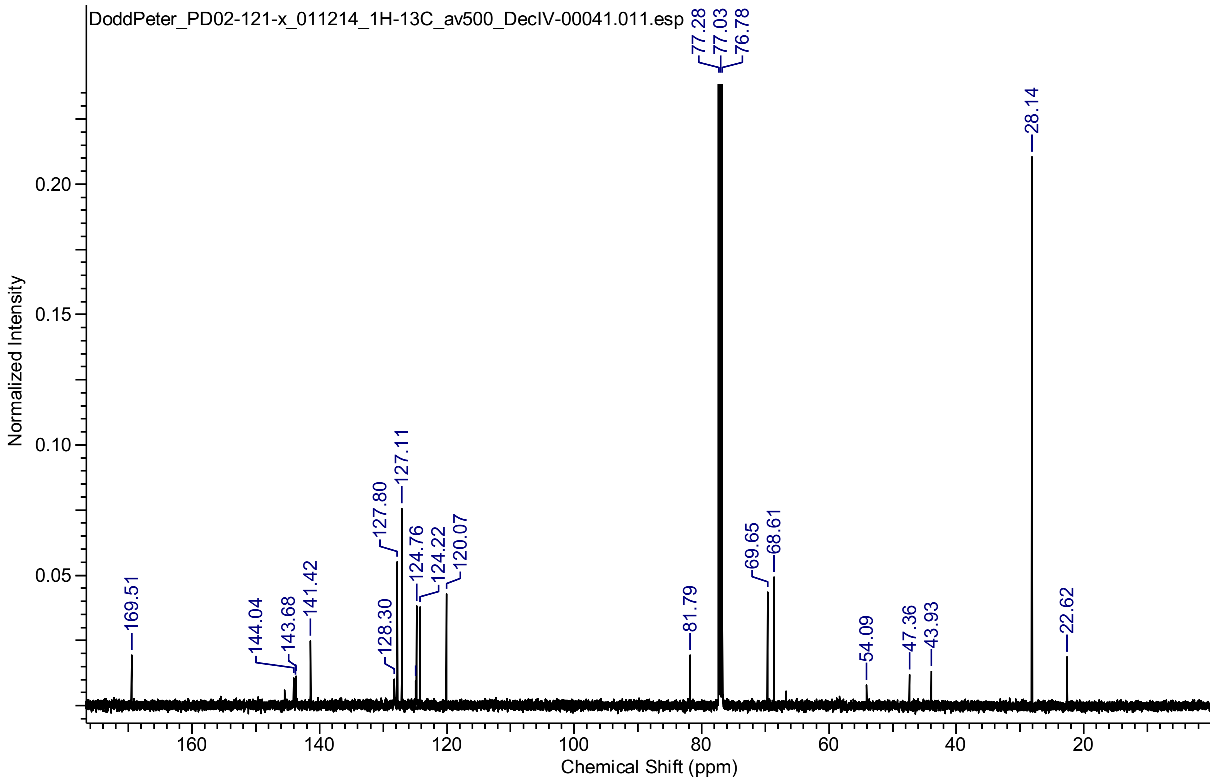

3-(carboxymethyloxy)propyl-[4-[(9H-fluoren-9-ylmethoxycarbonylamino)-[4-[methyl(oxido)sulfonio]phenyl]methyl]phenyl]-oxido-sulfonium (2)

^{1}H NMR

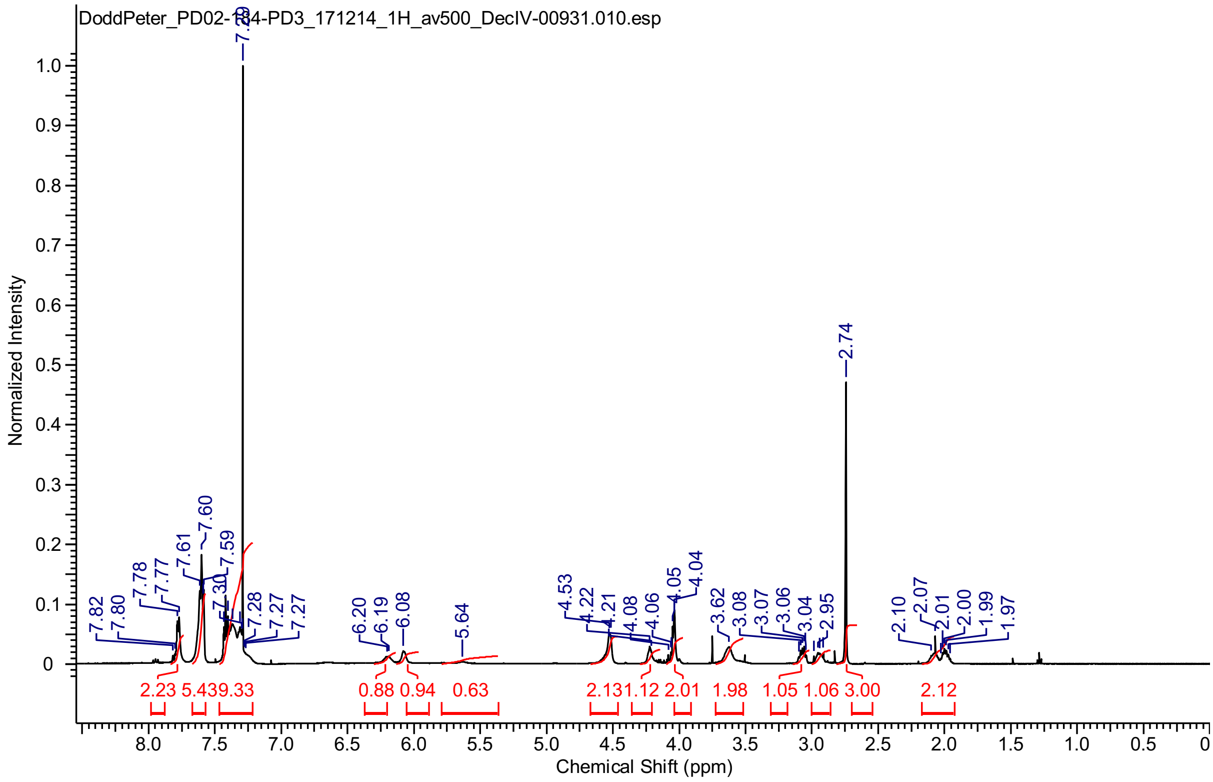

^{13}C NMR

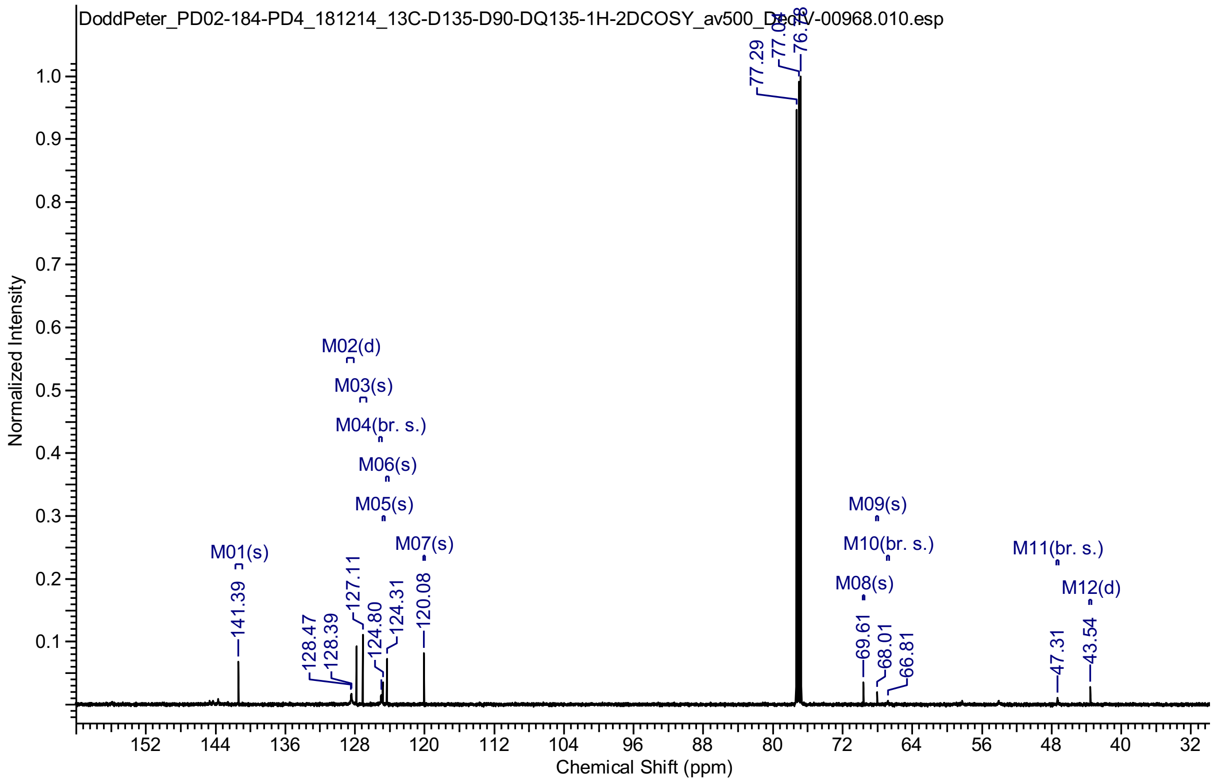
DoddPeter_PD02-184-PD4_181214_13C-D135-D90-DQ135-1H-2DCOSY_av500_DedV-00968.010.esp
Normalized Intensity
Chemical Shift (ppm)
M01(s)
M02(d)
M03(s)
M04(br. s.)
M05(s)
M06(s)
M07(s)
M08(s)
M09(s)
M10(br. s.)
M11(br. s.)
M12(d)
141.39
128.47
128.39
127.11
124.80
124.31
120.08
77.29
69.61
68.01
66.81
47.31
43.54
